# Detection of Foreign Bodies by Spiral Computed Tomography and Cone Beam Computed Tomography in Maxillofacial Regions

**DOI:** 10.5681/joddd.2014.030

**Published:** 2014-09-17

**Authors:** Farzaneh Kaviani, Reza Javad Rashid, Zahra Shahmoradi, Masoud Gholamian

**Affiliations:** ^1^Assistant Professor, Department of Oral and Maxillofacial Radiology, Faculty of Dentistry, Tabriz University of Medical Sciences, Tabriz, Iran; ^2^Assistant Professor, Department of Radiology, Faculty of Medicine, Tabriz University of Medical Sciences, Tabriz, Iran; ^3^Assistant Professor, Department of Oral and Maxillofacial Radiology, Faculty of Dentistry, Birjand University of Medical Sciences, Birjand, Iran; ^4^Post-graduate Student, Department of Radiology, Faculty of Medicine, Tabriz University of Medical Sciences, Tabriz, Iran

**Keywords:** Cone-beam computed tomography, foreign body, spiral computed tomography

## Abstract

***Background and aims.*** The imaging techniques commonly used for foreign body detection include plain radiography, xeroradiography, computed tomography (CT) scans, magnetic resonance imaging (MRI) and ultrasonography. The aim of the present study was to compare cone-beam computed tomography (CBCT) with conventional CT scan in determination of the exact location of a foreign body in the maxillofacial area in vitro.

***Materials and methods.*** In this descriptive study, seven different materials were selected as foreign bodies with dimensions of approximately 2 mm, 1 mm, and 0.5 mm. These materials consisted of metal, glass, wood, stone, plastic, graphite and tooth. These foreign bodies were placed in a sheep head between the corpus of the mandible and muscle, in the tongue and in an air space. One conventional CT scan and two CBCT scans were made on the models.

***Results.*** Tooth, metal, stone and glass foreign bodies were seen clearly on CT and CBCT scans made by NewTom at the smallest size in air. However, CBCT scan by NewTom was a more effective technique for visualization of foreign bodies in air compared to conventional CT. Foreign bodies measuring 0.5 mm made of metal, stone, glass, graphite and teeth were detected by all devices in muscle tissue and adjacent bone.

***Conclusion.*** According to the results, CBCT scans of NewTom and Planmeca are appropriate tools for detecting foreign bodies with relative high density in the maxillofacial area.

## Introduction

Foreign bodies usually enter the head and neck areas due to trauma or medical interventions. Pain, inflammation, intracranial abscesses and impaired wound healing are problems caused by foreign bodies. The location and the composition of a foreign body can vary greatly based on the type of trauma.^1-3^ Identification and localization of foreign bodies are based on history and clinical and radio-graphic examinations. Determination of the exact location of a foreign body is very important, especially when it is adjacent to vessels. In these cases, removal is associated with high risks for patients.^4,5^ Most foreign bodies are pieces of metal, wood and glass.^2^ The imaging techniques used for foreign body detection include plain radiography, xeroradiography, computed tomography (CT) scan, magnetic resonance imaging (MRI) and ultrasonography.

Plain radiographs are usually the first examination in detection of foreign bodies.^6-10^ In cases where the object would not be detected on plain radiographs or there is a need to identify the exact location of the object, CT scans will be helpful. Since CT scans are multi-planar and have high contrast, this method is the gold standard in detection of foreign bodies.^5^

Cone-beam computed tomography (CBCT) is a new imaging technique for maxillofacial imaging, with many advantages over conventional CT technique, including lower radiation dose, lower cost and sub-millimeter resolution. Compared to conventional CT scanning, CBCT is much less time-consuming and the time needed in for the procedures is usually less than 30 seconds. Despite enormous interest in CBCT, this technique has limitations including the geometric projection of cone beam, sensitive detectors and contrast resolution.^11^ Due to the limitations in resolution and efficacy of the CBCT images over CT scans, few studies have investigated the diagnostic power of CBCT in detection of foreign bodies.^12^ In the few available studies, the ability of CBCT in the diagnosis of metallic foreign bodies and metal artifacts was reported to be lower than that of CT.^13-15^ The results of a previous study showed the ability of digital volumetric tomography (DVT) in detection of foreign bodies in air and muscle tissue, where an air box and cow tongue muscle were used for reconstruction of space around the object and the influence of surrounding tissues and scattered radiation associated with the power system contrast resolution was ignored.^1-3^

In this descriptive study, CBCT was compared with conventional CT scan by placing different foreign bodies in three maxillofacial spaces in different sizes, using different CBCT devices. An *in vitro* model containing the tissues of living models was used for muscle tissue and adjacent bony tissue. The sinus area was reconstructed with empty boxes in previous studies while in the present study a dry human skull was used to evaluate the attenuation effect of bony walls on the foreign body.

## Materials and Methods

### Samples and Foreign Bodies

Head of a sheep was used as a sample in this study. For each material a head was considered (seven heads of sheep). First, an initial scan of the head was carried out to rule out foreign bodies and anomalies. The prepared samples were used one day after death, and all the images were taken on the same day.

To mimic foreign bodies in the muscle tissue, a foreign body was placed on the tongue muscle of sheep. Ventral body of the tongue was developed using a vertical cutting blade and the foreign body was pushed into the muscle.

To mimic foreign bodies in the adjacent bone, foreign bodies were placed between the mandibular cortex and the adjacent muscle. With a scalpel, a slot was prepared in the muscle and the foreign body was placed vertically on the surface of bone.

To mimic foreign bodies in air, a foreign body was put into a dry skull of the human.

Seven different most common foreign bodies found in the head and neck areas were chosen, including metal, glass, plastic, stone, wood, graphite and teeth. Each material was prepared in three sizes: 2 mm, 1 mm, and 0.5mm. Hounsfield unit (HU), radiopacity of foreign bodies and their surroundings were measured by spiral CT images (Table 1 ).

**Table 1:** Radiopacity of the investigated foreign bodies and their surrounding tissues according to Hounsfield unit (HU) scale

Material	Hounsfield Unit
Metal	4000
Glass	2407
Wood	60
Stone	1876
Acrylic resin	193
Graphite	742
Tooth	1881
Cortical bone	1949
Muscle	71
Air	-932

### Imaging

Samples were scanned by one CT scan device (Emotion 16 Spiral CT, Somatom Sensation 16, Siemens, Forcheim, Germany) and two CBCT devices (Planmeca Cone Beam CT, Helsinki, Finland; and NewTom VG Cone Beam CT scan, Verona, Italy).

**Figure 1.**
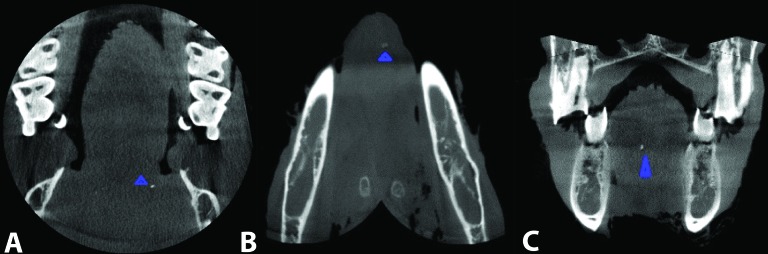
(A) Foreign body in Spiral CT. Glass measuring 0.5 mm in size in muscle. (B) Foreign body in Spiral CT. Graphite measuring 1 mm in size in muscle. (C) Foreign body in Spiral CT. Plastic measuring 2 mm in size in muscle.

Somatom Sensation 16 was used, with a matrix size of 512 × 512 and 0.4 mm resolution and 140 kVp. Scanning was carried out at kVp = 110 and MA = 110 and a minimum thickness of 0.6 mm was used for this evaluation. The reconstruction was performed using Syngo CT 2009E software and was assessed with Leonardo Work Station (Figure 1).

**Figure 2.**
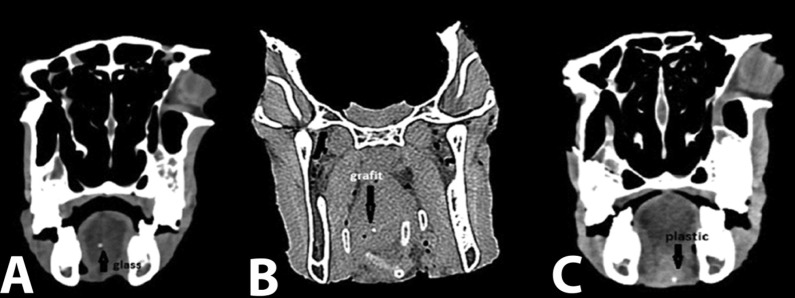
(A) Foreign body in NewTom CBCT; metal measuring 0.5 mm in sizeon cortical bone. (B) Foreign body in NewTom CBCT; graphite measuring 1 mm in size in muscle. (C) Foreign body in NewTom CBCT; glass measuring 0.5 mm size in muscle.

NewTom VG Cone Beam CT was used, with cone x-ray beam and 1920 × 1536-pixel flat panel detector, 15 × 15 cm detector size, 360 degree rotation, 18 s scan time and 120 kVp. The slices were prepared at kVp = 110 at a scan time of 18s and a minimum thickness of one millimeter. Initial and final reconstruction was performed using NNT Viewer software version 2.17 (Figure 2).

**Figure 3.**
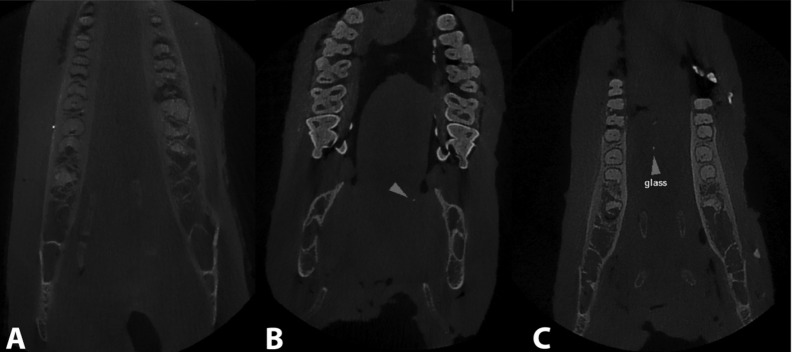
(A) Foreign body in Planmeca CBCT;graphite measuring 1 mm in size in muscle. (B) Foreign body in Planmeca CBCT; plastic measuring 2 mm in size in muscle. (C) Foreign body in Planmeca CBCT; glass measuring 1 mm in size in muscle.

Planmeca Promax 3D Max Cone Beam CT was used with detector flat panel pixels of 1516×1900, detector size of 8 × 8 cm, voxel size of 160 micrometers, spin degree of 270, a maximum scan time of 17s and kVp of 84. The scans were performed at kVp = 84 and mA = 16, and a minimum thickness of 1 millimeter was used. Initial reconstruction was performed by Romexis 2.3.1 software (Figure 3).

The images were observed by three radiologists, two maxillofacial radiologists and one general radiologist, who were aware of the foreign body. The observers expressed their opinion about the foreign bodies in images using the basic criteria described in Table 2.^2^

**Table 2:** Basic criteria used for image interpretation

Grade	Assessment	Definition
++++	Excellent	Excellent resolution of details and excellent visibility, good demarcation from surrounding
+++	Good image	Good resolution of details, demarcation from surrounding, clear visibility
++	Fair image	Insufficient resolution of detail, insufficient visibility, insufficient demarcation
+	Bad image	Details not resolved, bad demarcation from surrounding, bad visibility
0	No image	Invisible

## Results

Kappa coefficient of agreement between the observers was considered high (0.8).

### Air

Tooth, metal, stone, and glass foreign bodies were easily detected in CT and NewTom images at the smallest size. Teeth, metal and glass were clearly observed on images produced by Planmeca device at the size of 0.5 mm, but stone was not clearly seen with this size. All of the foreign bodies measuring 1 and 2 mm were detected on all scans. NewTom showed the objects with better details. Smallest sizes of wood, plastic and graphite were not detected on images of the tested devices.

### Muscle Tissue

Foreign bodies, 0.5 mm in size, made of metal, stone, glass, graphite, and teeth were detected on all scans. All the objects, 1 mm in size, except wood were detected on all scans. However, 1-mm wood and plastic objects could not be observed on NewTom images. Objects with a size of 2 mm, except for wood, were detected on all scans. By comparison, objects seen on CT images, followed by those of Planmeca, exhibited better detail.

### Adjacent Bone

Foreign bodies, 0.5 mm in size, made of metal, stone, glass, and graphite were detected well on all scans; 0.5-mm-in-size graphite was detected only by CT scan. Metal, stone, glass, tooth, and graphite measuring 1 and 2 mm were detected on all scans. Wood was not detected in any of the scans. Also, plastic was not detected on NewTom images in any size (Table 3).

**Table 3:** The smallest size of foreign body in millimeters as detected by computed tomography (CT) and two cone beam computed tomography (CBCT) devices (NewTom and Planmeca)

Materials	Air			Muscle			Adjacent bone		
	CT	NewTom	Planmeca	CT	NewTom	Planmeca	CT	NewTom	Planmeca
Metal	0.5	0.5	0.5	0.5	0.5	0.5	0.5	0.5	0.5
Teeth	0.5	0.5	0.5	0.5	0.5	0.5	1	1	1
Wood	1	1	1	—	—	—	—	—	—
Plastic	1	1	1	1	2	1	1	—	1
Stone	0.5	0.5	1	0.5	0.5	0.5	0.5	0.5	0.5
Glass	0.5	0.5	0.5	0.5	0.5	0.5	0.5	0.5	0.5
Graphite	1	1	1	0.5	0.5	0.5	0.5	1	1

## Discussion

The imaging technique used for detection of foreign bodies entering the body is dependent on the physics of imaging and the characteristics of the foreign body such as the material, size, and its location.^16,17^ The first method for the detection of foreign bodies is plain radiography. Each technique has its own restrictions, and an object that could not be detected on a certain scan might be detectable on other images. Due to the overlapping of the shadows of objects with similar density, some objects cannot be seen on plain radiographs. The same is true for objects in deeper locations and those with smaller sizes. Ultrasonography is also impossible for the objects adjacent to air or behind bony structures. In addition, artifacts produced adjacent to metal objects in CT or magnetic resonance imaging (MRI) are among the restrictions of these techniques.^18,19^ In addition to CT, DVT has been used for the detection of foreign bodies.^1,3^ While DVT was demonstrated to be a suitable technique in detecting foreign bodies, wood and resin objects could not be detected in muscle tissue. The minimum size of detectable object in air is not significantly different between CT and DVT, whereas more contrast is required for objects in muscle tissue.^1,3^

The results of the present study showed that CT produces clearer images in muscle tissues and adjacent bony structures, while NewTom produces clearer images for objects in air (stone and teeth). Also, the images provided by Planmeca in muscle tissue and adjacent to bone were more similar to CT images.

Aras et al^2^ concluded that metal, glass and stone could be seen on all areas of plain radiographs, CT images and ultrasonography and objects with less radiopacity were seen in CT imaging. CT demonstrated a better function compared to ultrasonography and plain radiography in detecting foreign bodies of the sinuses.^2^

In the present study, foreign bodies with high opacity were detected in air with both imaging techniques, but low-density objects could be detected with 1 mm in size and bigger sizes.

In this study, wood, small plastic, and graphite were not observed in adjacent bone either. As shown in previous studies, MRI is an alternative reliable device for detection of objects with low radiopacity.^4,12,18,19^

In general, the higher opacity of the object is associated with higher grey level, which results in a higher possibility of detection. Another factor that is involved in observing the object is spatial resolution that exhibits the ability of an imaging system to visualize an object with high contrast, which is limited in CT and CBCT with pixel size and voxel size, respectively.^11^

In the present study, the lower voxel size of CBCT, compared to the pixel size of CT, showed that CBCT has higher spatial resolution and greater ability to detect high-density foreign bodies.

The disadvantage of CBCT can be considered its limitation in detecting low-density objects. Radiopaque foreign bodies which could be seen in air with high clarity had ragged margins when placed in muscle tissue. The ability of NewTom and Planmeca devices was strongly confirmed in detecting foreign bodies with relatively high density.

Relatively low-density substances in muscle tissue and adjacent bone were detected by Planmeca better than NewTom because of smaller field and less scattered beam of the device, which can improve the contrast resolution of the images.

In the present study, the objects with very high density such as metallic objects could be detected by all the devices even in the smallest sizes but there were some limitations for surrounding artifacts in all devices. With regards to the studies that show CBCT can produce less artifacts compared to conventional CT, CBCT is able to present more detailed images of the dimensions of the foreign body and help in localization of it.^11^

The assessment of objects with low density revealed that objects like wood, plastic, and graphite cannot be detected in sinus area. Objects with similar density of adjacent cortical bone can hardly be detected in very small sizes, while these objects are detectable when placed in muscle tissue. In cases where HU of the object is similar to that of the surrounding issue, the device with higher contrast and resolution can detect more efficiently. In addition, in cases where the radiopacity of the foreign body is similar to that of the cortical bone, small foreign bodies cannot be distinguished from muscle and bony structures while these objects are detectable only in muscle tissue.

## Conclusion

It can be concluded from the results of the present study that CBCT with lower radiation dose and lower cost can be used for detecting foreign bodies and their localization in cases of limited access to CT scan. CT and CBCT scans are not suitable for low-density foreign bodies, and MRI or ultrasonography can be recommended in these cases.
